# SCARA3 inhibits cell proliferation and EMT through AKT signaling pathway in lung cancer

**DOI:** 10.1186/s12885-022-09631-z

**Published:** 2022-05-16

**Authors:** Jeeho Kim, Ho Jin You, Chakyung Youn

**Affiliations:** 1 Laboratory of Genomic Instability and Cancer therapeutics and Department of Pharmacology, Chosun University School of Medicine, 375 Seosuk-Dong, Gwangju, 501-759 South Korea; 2 Department of Pharmacology, Chosun University School of Medicine, 375 Seosuk-dong, Gwangju, 501-759 South Korea; 3 Department of Meridian & Acupoint∙Diagnosis College of Korean Medicine, Dongshin University 67, Dongsindae-gil, Naju-si, Jeollanam-do Republic of Korea

**Keywords:** SCARA3, Lung Cancer, Tumor growth, AKT signaling

## Abstract

**Background:**

Scavenger receptor class A member 3 (SCARA3) is decreased in prostate cancer and myeloma. However, functions of SCARA3 in various cancers remain unclear. In this study, we tried to evaluate the functional study of SCARA3 in lung cancer.

**Methods:**

The expression level of SCARA3 in the TCGA-database, lung cancer tissue microarray and lung cancer cells and the prognosis of lung cancer patients were measured. Lung cancer tissue microarray was analyzed pathologically using immunohistochemistry, and quantitative analysis of SCARA3 in normal lung cells and lung cancer cells was analyzed using western blot analysis. Survival curves for lung cancer patients were prepared with the Kaplan-Meier method. Migration and invasion of SCARA3 overexpressed lung cancer cells were determined using a Transwell chamber system. Proliferation of lung cancer cells was determined based on cell viability assay using cell culture in vitro and a tumorigenicity model of BALB/C nude mouse in vivo.

**Results:**

The expression of SCARA3 was abnormally reduced in TCGA-database, lung tissue microarray, and various lung cancer cells. However, overexpression of SCARA3 reduced the proliferation of lung cancer. The ability of SCARA3 to inhibit cancer cell proliferation was maintained even in vivo using a mouse xenograft model. In addition, overexpression of SCARA3 reduced migration and invasion ability of lung cancer cells and induced decreases of EMT markers such as β-catenin, vimentin, and MMP9. We aimed to prove the role of SCARA3 in the treatment of Lung cancer, and shown that the expression level of SCARA3 is important in cancer treatment using cisplatin. The enhancement of the effect of cisplatin according to SCARA3 overexpression is via the AKT and JNK pathways.

**Conclusions:**

This study confirmed an abnormal decrease in SCARA3 in lung cancer. Overexpression of SCARA3 potently inhibited tumors in lung cancer and induced apoptosis by increasing sensitivity of lung cancer to cisplatin. These results suggest that SCARA3 is a major biomarker of lung cancer and that the induction of SCARA3 overexpression can indicate an effective treatment.

**Supplementary Information:**

The online version contains supplementary material available at 10.1186/s12885-022-09631-z.

## Background

In the late 1970s, scavenger receptor (SR) was found to be able to bind to acetylated low-density lipoproteins (acLDLs) [1]. Scavenger receptors are classified into eight classes according to their structural similarity. Scavenger receptor class A (SR-A) includes SCARA1 (MSR), SCARA2 (MARCO), SCARA3 (CSR), SCARA4 (COLEC12), and SCARA5 (TESR) [2, 3]. SCARA1 and SCARA2 are highly expressed in macrophages and dendritic cells. SCARA4 are highly expressed in endothelial cells, granulocytes, and neutrophils. SCARA5 are known to be highly expressed in epithelial cells. However, SCARA3 expression pattern in various cells remains unclear [4–8] . Although SCARA3 is structurally similar to SCARA1–5, SCARA3 and SCARA4 do not contain an SRCR domain that recognizes lipoproteins. Accordingly, SCARA3 does not have the ability to bind to lipoprotein [8].

The SCARA3 gene is located on chromosome 8p21.1 involved in UV irradiation and oxidative stress [9]. SCARA3 deficiency can promote differentiation of bone marrow mesenchymal stem cells (BMSCs) and adipose tissue-derived mesenchymal stem cells (Ad-MSCs) into adipocytes [10, 11]. Methylation at the promoter region of SCARA3 in Type2 Diabetes mellitus (T2DM) is increased [12]. It is known that SCARA3 is downregulated in prostate cancer and myeloma, but upregulated in ovarian carcinoma [13–15]. However, the expression level of SCARA3 in lung cancer remains unclear. Thus, the objective of this study was to determine the expression and role of SCARA3 in lung cancer. We found that SCARA3 was downregulated in lung cancer and that such downregulation was associated with a poor prognosis. Overexpression of SCARA3 caused a decrease in the Epithelial-Mesenchymal Transition (EMT) ability of lung cancer and an increase in sensitivity to cisplatin through AKT and JNK pathways. These findings provide evidence for the functional role and clinical significance of SCARA3 in lung cancer, suggesting that SCARA3 could be a potential therapeutic target to treat lung cancer.

## Methods

### Cell culture

All cell lines were obtained from the American Type Culture Collection (ATCC, Manassas, VA, USA). There were grown according to standard protocols. Human lung cell lines IMR90 and WI38 and human MRC5 fibroblasts were cultured in MEM medium. There were used within 10 passages. Human lung cancer cell lines (HCC827, H23, H358, A549, H460, SK-MES-1, H1650, H1666, Calu-1, Calu-3, and H1299) were maintained with RPMI 1640 medium (Welgene, Seoul, Korea). All media were supplemented with 10% fetal bovine serum (FBS, Invitrogen, Carlsbad, CA, USA), 100 units/mL of penicillin, and 100 μg/mL of streptomycin (100_X_ Pen Strep solution, Invitrogen). All cells were cultured in a humidified incubator with 5% CO_2_ at 37 °C.

### Tissue microarray and immunohistochemistry

Tissue microarray of lung cancer tissues was performed using a tissue microarray kit BS04116 (US Biomax, Inc.; Rockville, MD, USA). Clinical staging was assessed based on the AJCC cancer staging system (8th Edition). Immunohistochemistry was performed utilizing anti-SCARA3 antibodies (NBP1–32130, 1:100; Novaus, Centennial, CO, USA) according to the manufacturer’s protocol. Briefly, antigen retrieval was performed using 1_X_ antigen retrieval buffer (pH 9.0; Abcam, Cambridge, England) in a cooling chamber (Biocare Medial, Pacheco, CA, USA). Sections were heated under pressure for 15 min, allowed to cool for 20 min, and equilibrated to ambient temperature under tap water. Endogenous peroxidase was blocked using 3% H_2_O_2_ solution before incubation with primary antibody at 4 °C overnight. Tissue sections were then incubated with HRP-conjugated secondary antibody for 1 hour at room temperature (RT) before visualization using DAB. Finally, sections were counterstained with Harris’s hematoxylin.

### Immunoblot analysis

For total protein extraction, cells were lysed in ice-cold M-PER mammalian protein extraction reagent (78,501, Thermo Fisher, Pittsburg, PA, USA) with protease inhibitor (Complete mini, Roche, Darmstadt, Germany) on ice for 10 min. Cells were broken by sonication and subsequently centrifuged at 13,000 rpm for 10 min at 4 °C. The supernatant was collected and protein concentration was determined with a Bradford protein assay. Proteins were separated by (6–12) % SDS-PAGE and transferred to polyvinylidene difluoride membranes (PALL life sciences, Washington, NY, USA). Membranes were blocked with blocking solution (5% skim milk in TBS-T (10 mM Tris-HCl, pH 7.4, 150 mM NaCl, 0.1% Tween-20)) at RT for 1 h followed by incubation with primary antibodies at 4 °C overnight. The membrane was cleaved prior to hybridization with the antibody. Primary antibodies were anti-SCARA3 (1:1000, NBP2–13286, NOVUS), anti-β-catenin (1:1500, 8480, Cell Signaling, Danvers, MA, USA), anti-Vimentin (1:1500, 5741, Cell Signaling), anti-MMP-9 (1:1500, 2270, Cell signaling), anti-Cleaved PARP (1:1500, 5625, Cell Signaling), anti-Cleaved caspase3 (1:1500, 9661, Cell Signaling), anti-phospho-Akt (1:1500, 9271, Cell Signaling), anti-Akt (1:1500, 9272, Cell Signaling), anti-phospho-SAPK/JNK (1:1500, 9251, Cell Signaling), anti-JNK1 (1:1500, 44-690G, Thermo Fisher), anti-Bcl-xL(1:1500, 2764, Cell Signaling), anti-Bax (1:1500, 2772, Cell Signaling), anti-Noxa (1:1500, OP180, CALBIOCHEM, Darmstadt, Germany), anti-GAPDH (1:8000, SC-47724, Santa Cruz, Dallas, TX, USA), anti-Flag M2 (1:200, F1804, Sigma-Aldrich), and anti-Actin (1:8000, MAB1501, Merck Millipore, Darmstadt, Germany). Membranes were washed three times with TBS-T buffer and incubated with horseradish peroxidase (HRP) conjugated secondary antibodies (1:4000, Jackson Laboratory, West Grove, PA, USA) at RT for 2 h. After rinsing with TBS-T buffer three times, membranes were treated with immobilon western chemiluminescent HRP substrate (P90720, Merck Millipore). Specific bands were visualized using a Luminescent image analyzer LAS-4000mini (Fujifilm Life Science, Stanford, CT, USA) to evaluate protein expression levels.

### Bioinformatics analysis of RNA-Seq data in TCGA

The TCGA mRNA expression of discovery set was transformed into log_2_ scale. TCGA datasets contained survival data with clinical information. TCGA survival curves were visualized using UCSC Xena browser (https://xena.ucsc.edu/) and GraphPad Prism software version 8.0.

### Quantitative real-time PCR

Total RNAs of cells cultured in 60 mm culture dishes for 24 h were isolated using Trizol (Invitrogen) and reverse transcribed into cDNAs using Reverse Transcriptase M-MLV (Takara, Mountain View, CA, USA) according to the manufacturer’s protocol. Real-time PCR analysis was performed using a SYBR green-based fluorescent method (SYBR premix Ex Taq kit, Takara) and an ABI 7500 Fast Real-time PCR System (Applied Biosystems, Foster City, CA, USA) with specific primers. Primers used for real-time PCR were as follows: GAPDH forward, 5′-TTC ACC ACC ATG GAG AAG GC-3′ and GAPDH reverse, 5′-GGC ATG GAC TGT GGT CAT GA-3′; SCARA3 forward, 5′-GAA TTG CAG GGA AGA CAG GG-3′ and SCARA3 reverse, 5′-GTA GAA GCT CTG GCT TCC TGG-3′. The quantity of SCARA3 transcripts was calculated based on the threshold cycle (Ct) using the ∆∆Ct method after normalization against the level of GAPDH as an internal control.

### Generation of stable flag-SCARA3 cells

H1299 and A549 cells were transfected with SCARA3-pcDNA3.1+/C-(K) DYK (Flag) vector (GenScript; Piscataway, NJ, USA) or control- pcDNA3.1+/C-(K)DYK (Flag) vector using Lipofectamine 2000 (Invitrogen). At 24 h post transfection, cells were expanded 1:20 into complete media containing 0.3 mg/mL neomycin. Selection with neomycin was usually completed within 2 to 3 weeks. Clones stably overexpressing Flag-SCARA3 were confirmed by Western blot analysis.

### Migration and invasion assays

Cellular migration of H1299-SCARA3 and H1299-control cells was determined using 24-well Transwell permeable Supports (3422, Corning, Kennebunk, ME, USA). Cellular potential for invasiveness was determined using 24-well Matrigel invasion chambers (354,480, Corning). Cells were seeded into upper inserts at 2 × 10^5^ cells in 300 μL serum-free RPMI 1640. Outer wells were filled with 700 μL RPMI containing 10% FBS as chemoattractant. Cells were incubated at 37 °C with 5% CO_2_ for 16 to 20 h, stained with 1% Crystal violet for 5 min, and washed twice with PBS. Non-migrated or non-invading cells were removed by swabbing the top layer. Invasive cells were observed and photographed under an optical microscope in three random fields. Cells were counted using ImageJ software.

### Immunofluorescence microscopic analysis

Cells were cultured for 24 h on cover slips coated with poly-L-lysine (Sigma–Aldrich, St. Louis, MO, US). These cells were washed with PBS, fixed in methanol for 5 min at RT, and incubated with blocking buffer (1% BSA in PBS). These cells were then incubated with anti-β-catenin antibody (1:200, 8480, Cell Signaling) and anti-Flag M2 (1:200, F1804, Sigma-Aldrich) at 4 °C overnight. After washing with PBS three times, cells were incubated with secondary antibody (Alexa Fluor 594 chicken anti-rabbit, 1:200, Invitrogen) in blocking buffer at RT for 2 h. Cells were then washed with PBS three times and mounted using Fluorescent Mounting Medium with DAPI (GBI Labs, Mukilto, WA, USA). Images were acquired using a Zeiss LSM Meta confocal microscope (Carl Zeiss, Weimar, Germany) with an LSM Meta software. Image contrast and brightness were adjusted using an LSM image browser.

### Cell growth assay

Cell growth was analyzed with an EZ-Cytox cell viability assay kit (EZ-3000, Dogen, Seoul, Korea). Briefly, 1 × 10^6^ cells in the presence or absence of 5 μM LY294002 (Sigma–Aldrich) were cultured in a 96-well plate for 24 to 72 h. After adding 10 μL of ten-fold solution, cells were incubated at 37 °C with 5% CO_2_ for 1 to 2 h. Cell growth rate was determined by measuring absorbance at 450 nm with a microplate reader at two time points (72 h and 24 h). It was calculated as OD_450_ at 72 h/OD_450_ at 24 h.

### Tumor sphere formation assay

Single colony-dissociated cells were seeded into 24-well plates with ultra-low attachment surface (3473, Corning) and further incubated at 37 °C with 5% CO_2_ in a humidified incubator for 12 days (d). After cell images were collected with an optical microscope, tumor-sphere diameter was measured with an iSolution Lite software.

### Tumor formation in nude mice

Mice used in this study were 5-week-old male BALB/c nude mice purchased from NARA Biotech (Seoul, Korea). They were housed in a pathogen free facility (SPF) and treated according to standard protocols and animal welfare regulations. Mice used in the experiment were supplied with sufficient water and feed in sterile cages. H1299 and A549 cells overexpressing SCARA3 or deficient in SCARA3 were harvested, resuspended in PBS, and then injected subcutaneously into the left and right flanks of the BALB/C nude mice (1 × 10^6^ cells per flank, *n* = 5 mice per group). The size of a visible tumor was measured every 3 to 4 d using micrometer calipers. Tumor volumes were calculated with the following formula: volume = 0.5a × b^2^, where a and b were the larger and the smaller tumor diameters, respectively. Mice were humanely sacrificed at 12 weeks after injection. Primary tumors were excised, immediately weighed, and fixed in 4% paraformaldehyde. Significant differences between groups were assessed by two-tailed paired two-way ANOVA using GraphPad Prism (GraphPad Software Inc., CA, USA). Animal experiments were performed in accordance with the guidance Chosun University Institutional Animal Care and Use Committee. ARRIVE guidelines (http://arriveguidelines.org) were followed.

### Cell viability assay

Cell viability was analyzed with an EZ-Cytox cell viability assay kit (EZ-3000, Dogen) utilizing 1 × 106 cultured cells treated with 30 μM cisplatin (Sigma–Aldrich) for 24 or 48 h. After adding 10 μL of ten-fold solution, cells were continuously incubated at 37 °C with 5% CO_2_ for 1 to 2 h. Cell viability was assessed with survival percent of each sample based on the OD_450_ ratio of before/after treatment with cisplatin (treated growth OD450/untreated growth OD450 × 100).

### Statistical analysis

All statistical analyses were performed with Student’s t-test, two-way ANOVA test, and Mann–Whitney test using GraphPad Prism (GraphPad Software Inc., CA, USA). Survival curves were plotted with the Kaplan–Meier method. All data are presented as mean ± standard deviation (SD). Statistically significant differences are indicated as follows: *, *p* < 0.05; **, *p* < 0.001; and ***, *p* < 0.0001.

## Results

### SCARA3 is downregulated in lung cancer

**Fig. 1:**
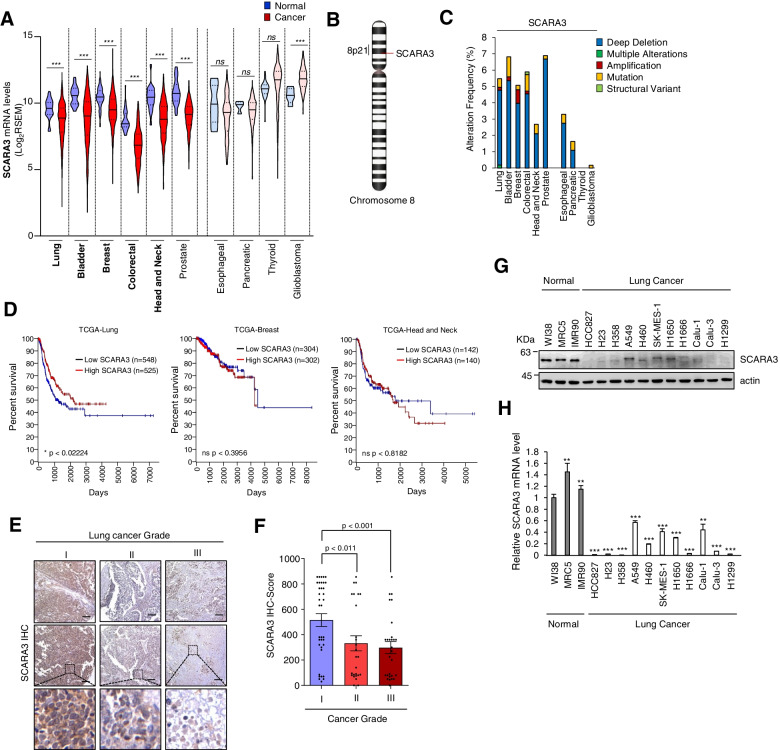
SCARA3 is aberrantly downregulated in lung cancer. **A** Expression of SCARA3 mRNA in 10 primary cancer types from TCGA. Data are shown as mean ± SD. ns, not significant; ***, *P* < 0.001, two-tailed Student’s t-test. **B** SCARA3 gene is located on the short arm of chromosome 8 at position 8q21.1. **C** Alteration frequency of SCARA3 gene in 10 cancer types from TCGA. **D** Kaplan-Meier analysis of survival according to SCARA3 in lung cancer, breast cancer, and head and neck cancer patients. *P* values are from a Log-rank test. **E, F** SCARA3 protein was immunohistochemically stained with brown color in the cytoplasm and/or nuclei of lung cancer tissues. Scale bar = 100 μm. **G, H** Expression levels of SCAR3 proteins (**G**) mRNAs (**H**) were determined by Western blot and RT- qPCR, respectively, using specific anti-SCARA3 antibodies and SCARA3-specific primers in various lung cancer cell lines and adjacent normal cell lines. Data are presented as mean ± SD of three independent experiments. **, *P* < 0.01; ***, *P* < 0.001

It has been reported that SCARA3 is downregulated in prostate cancer and myeloma [13, 14]. However, studies on the functional role of SCARA3 in cancer are lacking. We predicted that SCARA3 could also be down-regulated in other cancers such as lung cancer. To prove this, we analyzed SCARA3 mRNA levels in normal and cancer tissues of 10 cancers using the TCGA-database. SCARA3 was statistically and significantly downregulated in lung, bladder, breast, colon, head and neck cancers, including previously reported prostate cancer (Fig. 1A). The chromosomal 8p21 region, where tumor suppressors such as TRAIL-receptor and MTSG1 as well as the SCARA3 gene are located [14, 16–18], is frequently deleted in lung cancer (Fig. 1B). Therefore, we analyzed the alteration frequency of SCARA3 in the TCGA-database of various cancers. Chromosomal changes in the SCARA3 gene were found in 8 cancers except for thyroid and glioblastoma. Deep deletion was found to be the most frequent change (Fig. 1C). These results corresponded to Fig. 1A. Next, we analyzed the survival rate (progression-free interval) according to the expression level of SCARA3 in lung, breast, and head and neck cancer patients. In breast and head and neck cancers, SCARA3 was downregulated in cancer tissues compared to that in normal tissues (Fig. 1A). However, the expression level of SCARA3 had no significant effect on the survival rate of cancer patients (breast cancer, *p* < 0.3956; head and neck cancer, *p* < 0.8126). SCARA3 was also downregulated in lung cancer tissues compared to that in normal tissues. However, the low expression level of SCARA3 was significantly associated with a poor survival rate of lung cancer patients (*p* < 0.02224) (Fig. 1D). According to previous reports, overexpression of SCARA5 has an anti-cancer effect [19–22]. Therefore, we compared expression levels of all SCARA genes in lung cancer. Although all five SCARA family members were significantly downregulated in lung cancer tissues compared to those in normal lung tissues, only SCARA3 expression level in lung cancer tissues showed statistically significant relation with patient survival (SCARA1, *p* = 0.4195; SCARA2, *p* = 0.9921; SCARA3, *p* < 0.02224; SCARA4, *p* = 0.8694; SCARA5, *p* < 0.6959) (Supplementary Fig. S1). In addition, to investigate the clinical significance of SCARA3 in lung cancer specimens, tissue microarray (TMA) data were analyzed for lung cancer tissues composed of different grades of carcinoma. As lung cancer tumor grade increased, there was a gradual and significant decrease in SCARA3 expression level (Fig. 1E and F). Consistent with the TCGA-lung data and immunohistochemistry results, significantly lower levels of SCARA3 protein and mRNA were detected in lung cancer cell lines than in normal primary human diploid lung fibroblasts (WI38, MRC5, IMR90) (Fig. 1G and H). These results suggest that SCARA3 is abnormally downregulated in lung cancer and that it might be a potential tumor suppressor candidate.

### SCARA3 inhibits proliferation of lung cancer cells

**Fig. 2:**
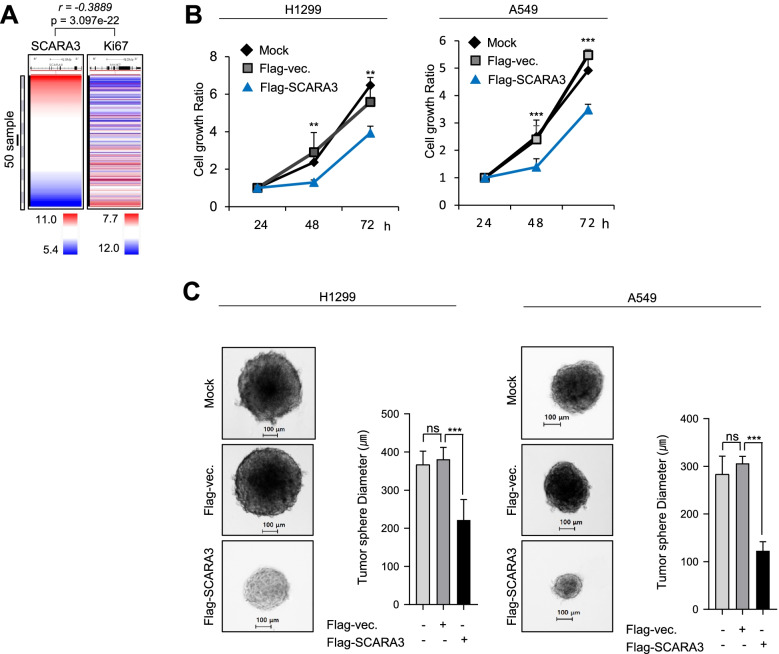
SCARA3 inhibits the growth of lung cancer. **A** Co-expression heat map of SCARA3 with Ki-67 in TCGA-LUAD (*n* = 706) derived from the UCSC Xena browser. **B** Cell proliferation was determined with an EZ-Cytox assay using H1299 and A549 cells at time points of 24, 48, and 72 h. Data are presented as mean ± SD of three independent experiments. ***, *P* < 0.001; **, *P* < 0.01. **C** Tumor sphere diameters in ultralow attachment plates were analyzed utilizing H1299 and A549 cells. Sphere formation was imaged on 12 d. Scale bars = 100 μm. Data are presented as mean ± SD of three independent experiments. *, *P* < 0.05; **, *P* < 0.01

**Fig. 3:**
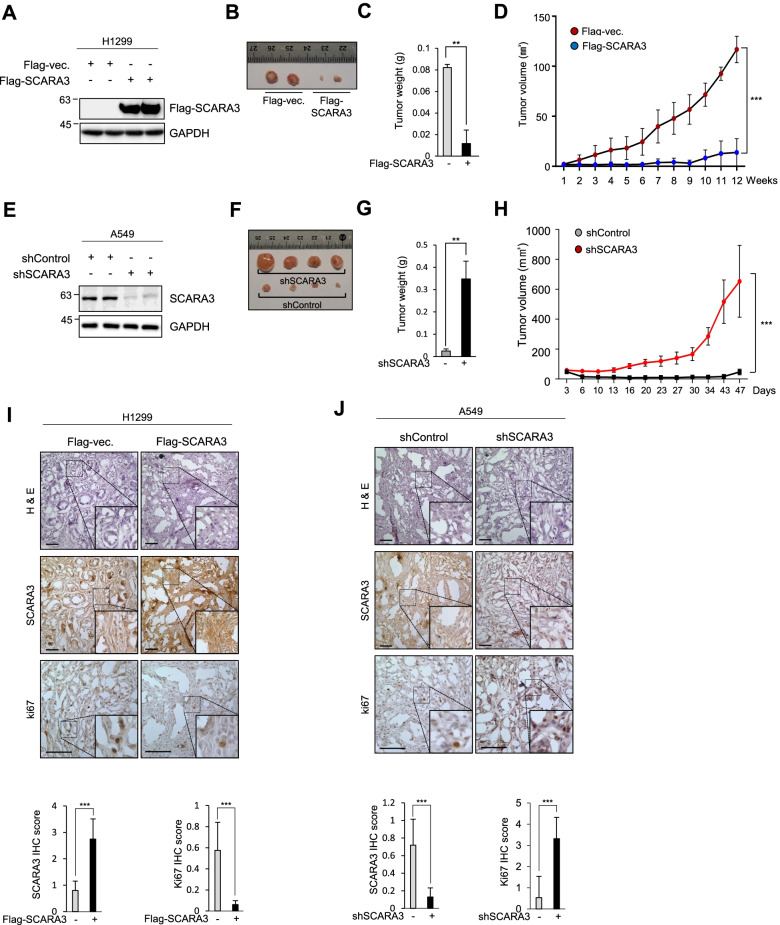
SCARA3 inhibits proliferation of lung cancer in vivo. **A** Western blot analysis for Flag-SCARA3 expression in indicated H1299 cells. **B** Tumors derived from indicated H1299 cells at end point (*n* = 5 mice per group). **C** Average tumor weight of indicated group at the endpoint of the experimenth. **D** Growth curves of tumors derived from indicated H1299 cells in mice. Data are shown as mean ± SD. ***, *P* < 0.001, two-way ANOVA. **E** Western blot analysis for shSCARA3 expression in indicated A549 cells. **F** Tumors derived from the indicated A549 cells at end point (*n* = 5 mice per group). **G** Average tumor weight of the indicated group at the endpoint of the experiment. **H** Growth curves of tumors derived from indicated A549 cells in mice. Data are shown as mean ± SD. ***, *P* < 0.001, two-way ANOVA. **I** and **J** Tumor sections from mice injected with indicated H1299 and A549 cells were stained with hematoxylin-eosin. Immunostained sections were stained with SCARA3 and Ki67. Scale bar = 100 μm

Considering that SCARA3 expression was decreased in lung cancer tissues and cells, SCARA3 expression might have an effect on lung cancer proliferation. To determine the role of SCARA3 in proliferation regulation, we analyzed RNA sequencing data from the TCGA-lung database and analyzed the correlation between the expression level of SCARA and a proliferation marker ki67. SCARA3 and ki67 were negatively correlated (*r* = − 0.3889, *p* < 3.097e-22) (Fig. 2A). The level of SCARA3 expression in lung cancer cells was comparatively lower than that in normal cells. In this study, SCARA3 protein was not detected in H1299 cells. It showed a low expression in A549 cells among 11 tested lung cancer cell lines (Fig. 1G). Thus, we analyzed cell growth rates of H1299 and A549 before and after the overexpression and/or deficiency of SCARA3. After overexpression of Flag-tagged-SCARA3 in H1299 and A549 lung cancer cells, cell growth rates were compared with those of the control cells. The overexpression of SCARA3 decreased the cell growth ratio by (1.56–1.72)-fold compared to the control group (Fig. 2B-C). Consistently, when tumor sphere formation analysis was performed, the tumor sphere diameter (mm^3^) was decreased by (1.76–2.61)-fold due to SCARA3 overexpression (Fig. 2C). In contrast, SCARA3 deficiency increased cell growth rate and tumor sphere formation in A549 (Supplementary Fig. S2A and Supplementary Fig. S2B). We tested whether the role of SCARA3 in inhibiting the proliferation of lung cancer was maintained not only in cell culture experiments conducted under artificial conditions, but also in an in vivo environment. Overexpression of Flag-tagged SCARA3 was induced in H1299 cells. These cells were then subcutaneously injected into BALB/C nude mice. Mice were observed for 12 weeks. When flag-tagged SCARA3 was overexpressed, xenograft tumor growth was significantly reduced compared to that of the control group. The tumor weight was reduced by 8-fold and the tumor volume was decreased by 3.3-fold (Fig. 3(A-D)). When xenograft tumor tissues were isolated and analyzed by immunohistochemistry, overexpression of SCARA3 protein decreased the expression level of proliferation-specific marker ki67 protein (Fig. 3I). In contrast, SCARA3 deficiency was induced by stably expressing SCARA3-shRNA in A549 cells (Fig. 1G) in which SCARA3 was expressed relatively high. In BALB/C nude mice, intradermal cells were injected in the same way as H1299 cells. Mice were then observed for 12 weeks. SCARA3 deficiency significantly increased xenograft tumor growth compared to the control. In the case of SCARA3 deficiency, tumor weight was increased by 12.1-fold and tumor volume was increased by 12.82-fold (Fig. 3(E-H)). Correspondingly, tumor tissues xenografted with SCARA3-deficient A549 cells showed increased ki67 protein (Fig. 3J). Taken together, these results suggest that SCARA3 is closely related to the proliferation of lung cancer cells and that overexpressing SCARA3 can inhibit lung cancer cell proliferation.

### Overexpression of SCARA3 reduces the metastatic capacity of lung cancer

**Fig. 4:**
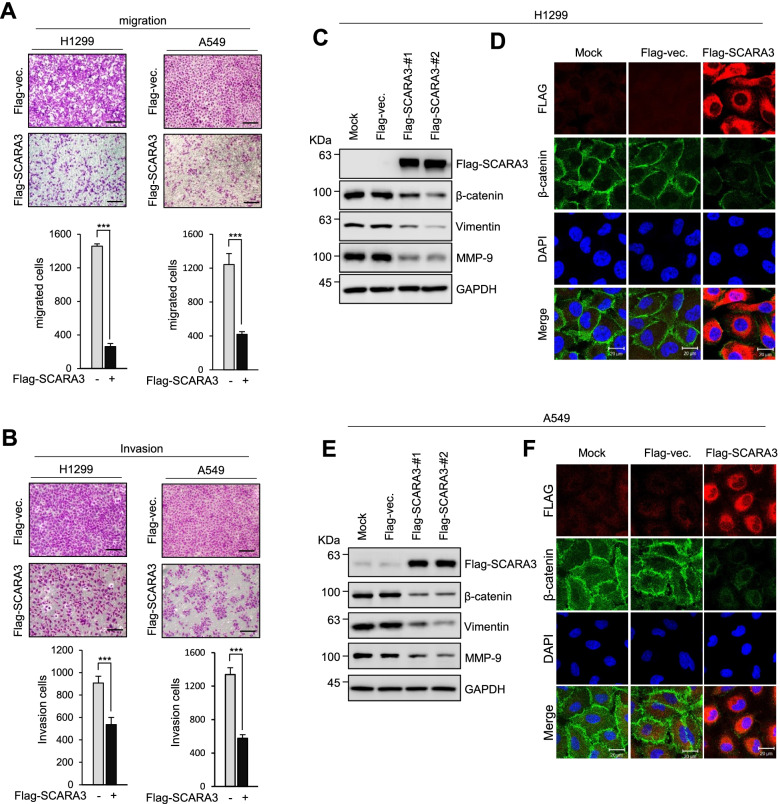
Overexpression of SCARA3 downregulates Epithelial-Mesenchymal Transition (EMT). **A** Migration of H1299 and A549 cells determined with Transwell assays. **B** H1299 and A549 invasion ability examined by Matrigel Transwell invasion assay. Quantitative results of migration and invasion assays are shown below. Data are presented as mean ± SD of three independent experiments. ***, *P* < 0.001. **C** Flag-tagged SCARA3 was expressed in H1299 cells. Protein levels of β-catenin, vimentin, and MMP9 were examined by Western blot using their specific antibodies. **D** Immunofluorescent staining of fixed H1299 cells with anti-Flag and β-catenin antibodies. Nuclei were stained with DAPI. Scale bar = 20 μm. **E** Flag-tagged SCARA3 was expressed in A549 cells. Protein levels of β-catenin, vimentin, and MMP9 were examined by Western blot using their specific antibodies. **F** Immunofluorescent staining of fixed A549 cells with anti-Flag and β-catenin antibodies. Nuclei were stained with DAPI. Scale bar = 20 μm

Metastasis to other organs is frequently observed in lung cancer patients, which is closely related to the survival rate and prognosis of patients [23, 24]. We predicted that overexpressing SCARA3 could reduce the ability of Epithelial-Mesenchymal Transition (EMT) in lung cancer. To prove this, migration and invasion abilities of lung cancer cells were analyzed using a Transwell chamber system. Results showed that migration and invasion abilities of H1299 and A549 cells overexpressing Flag-tagged-SCARA3 was significantly reduced compared to those of control cells (Fig. 4A and B). Conversely, migration and invasion capacities of A549 cells with depletion of SCARA3 were significantly increased compared to those of control cells (Supplementary Fig. S2C and Supplementary Fig. S2D). Next, we predicted that EMT marker protein could also change according to the overexpression of SCARA3. Therefore, we analyzed expression levels of EMT marker proteins such as b-catenin, vimentin, and MMP9 using western blot and immunochemistry. Expression levels of β-catenin, vimentin, and MMP9 proteins were significantly decreased in both H1299 and A549 cells overexpressing Flag-tagged SCARA3 (Fig. 4(C-F)). These findings suggest that high expression levels of SCARA3 in lung cancer may inhibit the metastatic ability of tumors.

### SCARA3 increases sensitivity of lung cancer to cisplatin

**Fig. 5:**
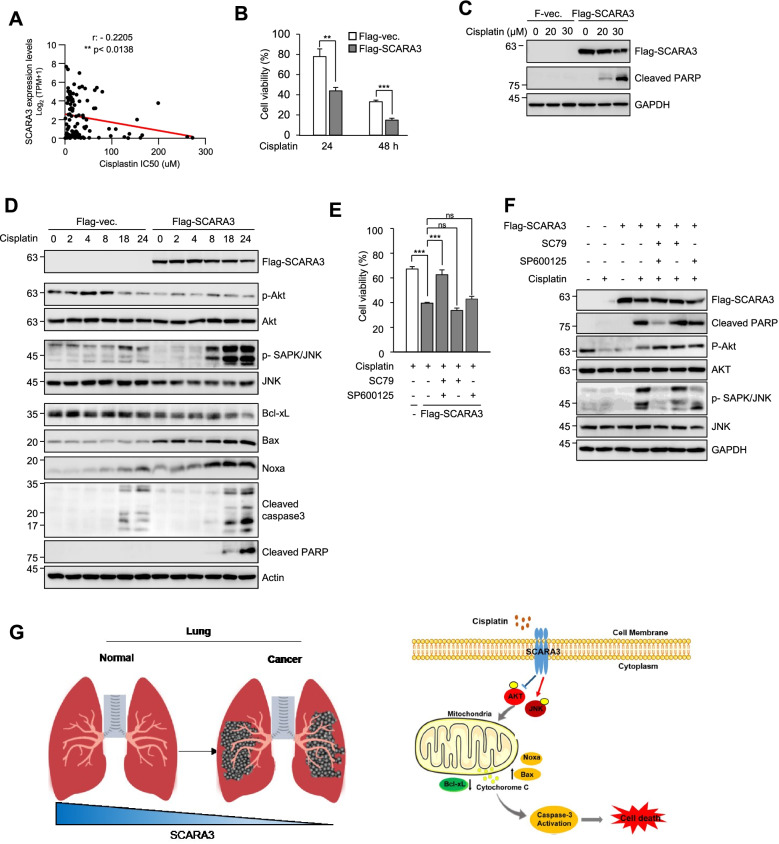
SCARA3 increases sensitivity of lung cancer cells to cisplatin via the AKT pathway. **A** Pearson’s correlation analysis showing half-maximal inhibitory concentration (IC_50_) values of cisplatin in lung cancer cell lines. **B** Cell viability was measured for H1299 cells overexpressed with empty vector or Flag-tagged SCARA3 followed by treatment with cisplatin (30 μM) for 24 or 48 h. Data are presented as mean ± SD of three independent experiments. **, *P* < 0.01; ***, *p* < 0.001. **C** Level of cleaved-PARP in H1299 cells overexpressed with empty-vector or Flag-tagged SCARA3 was analyzed by Western blot after treatment with indicated concentration of cisplatin for 24 h. **D** H1299 cells overexpressed with empty-vector or Flag-tagged SCARA3 were analyzed by Western blot using indicated antibodies after treatment with cisplatin (30 μM) for 0, 2, 4, 8, or 24 h. **E** Cell viability was measured for empty-vector or Flag-tagged SCARA3 overexpressed H1299 cells after treatment with SC79 (5 μM, AKT activator) and/or SP600125 (30 μM, JNK inhibitor) along with cisplatin (30 μM) treatment. Data are presented as mean ± SD of three independent experiments. ns, not significant; ***, *p* < 0.001. **F** Western blot analysis was performed for empty-vector or Flag-tagged SCARA3 overexpressed H1299 cells after treatment with SC79 (5 μM, AKT activator) and/or SP600125 (30 μM, JNK inhibitor) along with cisplatin (30 μM). **G** Schematic of a model for the main mechanism involved in the tumor suppression function of SCARA3 in lung cancer

Cisplatin is one of effective chemotherapeutic agents used for solid cancer. However, chemotherapeutic resistance to cisplatin is one of the major causes of treatment failure. Therefore, a more effective general-purpose treatment strategy is needed to improve clinical outcomes [25, 26]. We hypothesized that the expression level of SCRA3 in lung cancer could be related to resistance to cisplatin. We analyzed RNA sequence data from the Cancer Cell Line Encyclopedia (CCLE) RNA-Sequencing database and half-maximal inhibitory concentration (IC50) data from the Genomics of Drug Sensitivity in Cancer (GDSC) database. We found a negative correlation of SCARA3 expression with cisplatin IC_50_ in lung cancer cells, which was statistically significant (*r* = − 0.2205, *p* < 0.0138) (Fig. 5A). Compared with other lung cancer cells, H1299 cells exhibited cisplatin resistance and markedly reduced SCARA3 expression (Fig. 1G and H) [27]. To prove our hypothesis, H1299 cells were treated with cisplatin (30 μM) for 24 or 48 h along with overexpression of Flag-tagged SCARA3. Compared with control cells, when Flag-tagged SCARA3 was overexpressed, cisplatin treatment reduced cell viability by more than 2-fold (Fig. 5B). Consistently, when H1299 cells overexpressing Flag-tagged SCARA3 were treated with increasing concentration of cisplatin, cleaved-PARP, an apoptosis marker, was increased based on western blot analysis (Fig. 5C). These results suggest that increasing SCARA3 expression can decrease resistance of lung cancer cells to cisplatin.

### SCARA3 promotes apoptosis through AKT and JNK pathways

Since AKT is associated with chemotherapy resistance to cisplatin [27–30], we analyzed the correlation between cisplatin IC50 and AKT (AKT 1–3) or JNK (MAPK8) using CCLE-RNA sequencing data and GDSC data. The correlation between mRNA expression level of AKT and IC_50_ (uM) of cisplatin in various lung cancer cell lines (*n* = 101) was not statistically significant (Supplementary Fig. S3 (A-C)). However, the correlation between the mRNA expression level of JNK and the IC_50_ (uM) of cisplatin was statistically significant (*r* = 0.2223, *p* = 0.0262). The correlation between SCARA3 expression level and cisplatin IC_50_ in 11 tested lung cancer cell lines was not significant (*r* = − 0.5889, *p* = 0.0623) by Pearson’s correlation analysis. The correlation between the expression level of MAPK8 and cisplatin IC_50_ was not significant either (*r* = 0.0001618, *p* = 0.9997) (Supplementary Fig. 3D and Supplementary Fig. 3E). Therefore, we hypothesized that the increase in cisplatin resistance according to the decrease of SCARA3 was related to the activation of AKT and JNK rather than the expression level of AKT or JNK. To prove this, we overexpressed Flag-tagged SCARA3 in H1299 cells and treated there cells with cisplatin (30 μM) for 0 to 24 h. AKT and apoptosis-related proteins were then subjected to western blot analysis. When cisplatin was used to treat H1299 cells with SCARA3 overexpression, phosphorylation levels of AKT and anti-apoptotic protein Bcl-xL were decreased compared to those in the control group. In contrast, phosphorylation of JNK and apoptotic markers such as Bax, Noxa, cleaved caspase3, and cleaved PARP were increased (Fig. 5D). We tried to cross-check the decreased phosphorylation of AKT and the increase of phosphorylation of JNK as possible causes of increased sensitivity to cisplatin following SCARA3 overexpression. H1299 cells overexpressing flag-tagged SCARA3 were treated with AKT activator (SC79, 5 μM) and/or JNK inhibitor (SP600125, 30 μM) and then cell viability was analyzed. H1299 cells with SCARA3 overexpression showed reduced cell viability compared to control H1299 cells transfected with empty-vector. However, when H1299 cells with SCARA3 overexpression were co-treated with AKT activator and JNK inhibitor, cell viability recovered to control level (Fig. 5E). Correspondingly, after co-treatment with AKT activator and JNK inhibitor, the amount of cleaved PARP protein increased by SCARA3 overexpression also recovered to the control level based on western blot analysis (Fig. 5F). Taken together, these results suggest that SCARA3 can induce increased sensitivity of lung cancer to cisplatin via the AKT/JNK pathway.

## Discussion

In the present study, we confirmed that SCARA3 is a negative regulator of proliferation in lung cancer. Using TCGA-data analysis, CCLE-RNA sequencing and GDSC data analysis, tissue microarray, cell systems, and xenograft model, we conclude that SCARA3 plays a major functional and clinical role in lung cancer.

First, we focused on SCARA3 being downregulated in lung cancer and located in the chromosomal region 8p21. When a genomic alteration of SCARA3 gene was analyzed in the TCGA-data of various cancers, the frequency of deep deletion was relatively high in lung, bladder, breast, colorectal, hand and neck, and prostate cancers where SCARA3 was downregulated in cancer tissues compared to that in normal tissues. In chromosomal region 8p21, tumor suppressor genes such as MTSG1, BNIP3L, and BIN3 are located as well as Tumor necrosis factor-Related Apoptosis-Inducing Ligand (TRAIL) receptor gene cluster [14, 16, 31–34]. Deletion of this region is often observed in cancer cells associated with reduced sensitivity of tumor cells to anticancer drugs [17, 18, 35, 36]. From this point of view, our results can infer that SCARA3 might be a tumor suppressor candidate in lung cancer.

The second result we noted is the role of SCARA3 in cancer cell proliferation and metastasis ability. In the TCGA-Lung database, it was found that the mRNA expression of SCARA3 and the proliferation marker ki67 were negatively correlated. SCARA3 overexpression inhibited lung cancer cell proliferation. Inhibition of cancer cell proliferation caused by SCARA3 overexpression using a xenograft model was maintained in an in vivo environment. In cancer patients, the size of cancer and metastasis to other organs are two of the most important factors that determine the survival rate and prognosis of cancer patients [23, 24]. Interestingly, SCARA3 overexpression reduced the migration and invasion ability of lung cancer cells and induced a decrease in EMT marker protein. Our results are very important in that our results show that SCARA3 can both reduce the size of cancer and inhibit the metastasis ability [19–22].

Cisplatin is one of the most effective chemotherapeutic agents widely used for cancer treatment. However, resistance to cisplatin is a major cause of chemotherapy failure. Therefore, effective general-purpose treatment is strategically needed to improve clinical outcomes. AKT plays an important role in the process of acquiring resistance to chemotherapy drugs, including cisplatin [30, 37–42]. When cisplatin is administered, the apoptosis pathway is activated while the phosphorylation of AKT is reduced. However, in cisplatin-resistant cells, AKT phosphorylation is not reduced [27, 29]. We found that in H1299 cells, a cisplatin-resistant cell line, SCARA3 overexpression with cisplatin treatment induced a decrease in AKT phosphorylation. These results demonstrate that the expression of SCARA3 is comparatively low in cisplatin-sensitive A549 cells. In contrast, the SCARA3 expression was not detected in cisplatin-resistant H1299, Calu-1, or H358 cells (Fig. 1G). As such, we found that SCARA3 overexpression increased the sensitivity of lung cancer to cisplatin treatment.

Finally, based on the report that the SCARA3 promoter region is hyper-methylated in prostate cancer [13], there is a possibility that hyper-methylation is also present in lung cancer. In addition, sumoylation of the K582 region of SCARA3 can reduce protein stability [43]. Therefore, there is a need to analyze SCARA3 promoter methylation and study sumoylation of SCARA3 protein in lung cancer treatment using SCARA3 expression regulation.

## Conclusions

This study confirmed that SCARA3 was downregulated in lung cancer. Overexpression of SCARA3 inhibited lung cancer proliferation, reduced metastatic capacity, and decreased EMT marker proteins. These results suggest that SCARA3 has a strong tumor suppressor function in lung cancer. In addition, SCARA3 overexpression increased the sensitivity of lung cancer to cisplatin and induced death of lung cancer cells (Fig. 5G). Taken together, our results indicate that SCARA3 is a major biomarker of lung cancer and that the induction of SCARA3 overexpression in cisplatin-resistant lung cancer patients might be an effective treatment strategy.

## Supplementary Information

**Additional file 1: Fig. S1.** The expression level of SCARA3 in lung cancer is associated with poor prognosis. A. Expression of SCARA family members (SCARA1–5) mRNA in the TCGA-Lung database. Data are shown as the mean ± SD. ns, not significant; ***P < 0.001, two-tailed Student’s t-test. B. Kaplan–Meier analysis of survival according to the SCARA family members in lung cancer patients. *P* values are for a Log-rank test. ns, not significant; *P < 0.05. **Fig. S2.** SCARA3 increases tumorigenesis in Lung cancer. A. The cell proliferation was determined by EZ-Cytox in A549 cells at the time points of (24, 48, 72 and 96) h. The data are presented as the mean ± SD of three independent experiments. *** *P* < 0.001. B. Tumor sphere diameters in ultralow attachment plates were analyzed utilizing A549 cells. Sphere formation was imaged on 6 d. Scale bars = 100 μm. The data are presented as the mean ± SD of three independent experiments. *P < 0.05, **P < 0.01. C. The migration of A549 cells was determined using transwell assays. D. The A549 invasion ability was examined by Matrigel transwell invasion assay. The quantitative results of migration and invasion assays are shown below. The data are presented as the mean ± SD of three independent experiments. ***P < 0.001. **Fig. S3.** Genomics of Drug Sensitivity in Cancer (GDSC) database analysis. A-D. Person’s correlation analysis showing the half-maximal inhibitory concentration (IC50) values of cisplatin in lung cancer cell lines. (A) AKT1, (B) AKT2, (C) AKT3, and (D) MAPK8. ns, not significant; *P < 0.05. E. SCARA3, MAPK8 expression levels and cisplatin IC50 values were analyzed in 11 lung cancer cell lines using the CCLE-RNA sequencing database and the GDSC database.

**Additional file 2.**

**Additional file 3: Supplementary Table 1.** SCARA3 mRNA analysis in the TCGA-Lung database associated with Fig. 1a.

**Additional file 4: Supplementary Table 2.** Analysis of SCARA1–5 mRNA in the TCGA-Lung database associated with Supplementary Fig. 1a.

## Data Availability

All data needed to evaluate the conclusion in the paper are present in the paper and/or the Supplementary Materials. Figures 1A-D and 2A analyzed TCGA-database. The analyzed TCGA-data was added to Supplementary Materials (Supplementary Table 1 and Supplementary Table 2).
