# Supplementary material for: SCARA3 inhibits cell proliferation and EMT through AKT signaling pathway in lung cancer
Source: BMC Cancer. 2022 May 16;22:552. doi: 10.1186/s12885-022-09631-z (PMC9112459; doi:10.1186/s12885-022-09631-z)
Supplement: Supplementary file 1 — Additional file 1: Fig. S1. The expression level of SCARA3 in lung cancer is associated with poor prognosis. A. Expression of SCARA family members (SCARA1–5) mRNA in the TCGA-Lung database. Data are shown as the mean ± SD. ns, not significant; ***P < 0.001, two-tailed Student’s t-test. B. Kaplan–Meier analysis of survival according to the SCARA family members in lung cancer patients. P values are for a Log-rank test. ns, not significant; *P < 0.05. Fig. S2. SCARA3 increases tumorigenesis in Lung cancer. A. The cell proliferation was determined by EZ-Cytox in A549 cells at the time points of (24, 48, 72 and 96) h. The data are presented as the mean ± SD of three independent experiments. *** P < 0.001. B. Tumor sphere diameters in ultralow attachment plates were analyzed utilizing A549 cells. Sphere formation was imaged on 6 d. Scale bars = 100 μm. The data are presented as the mean ± SD of three independent experiments. *P < 0.05, **P < 0.01. C. The migration of A549 cells was determined using transwell assays. D. The A549 invasion ability was examined by Matrigel transwell invasion assay. The quantitative results of migration and invasion assays are shown below. The data are presented as the mean ± SD of three independent experiments. ***P < 0.001. Fig. S3. Genomics of Drug Sensitivity in Cancer (GDSC) database analysis. A-D. Person’s correlation analysis showing the half-maximal inhibitory concentration (IC50) values of cisplatin in lung cancer cell lines. (A) AKT1, (B) AKT2, (C) AKT3, and (D) MAPK8. ns, not significant; *P < 0.05. E. SCARA3, MAPK8 expression levels and cisplatin IC50 values were analyzed in 11 lung cancer cell lines using the CCLE-RNA sequencing database and the GDSC database. [file 12885_2022_9631_MOESM1_ESM.pptx]

## Slide 1
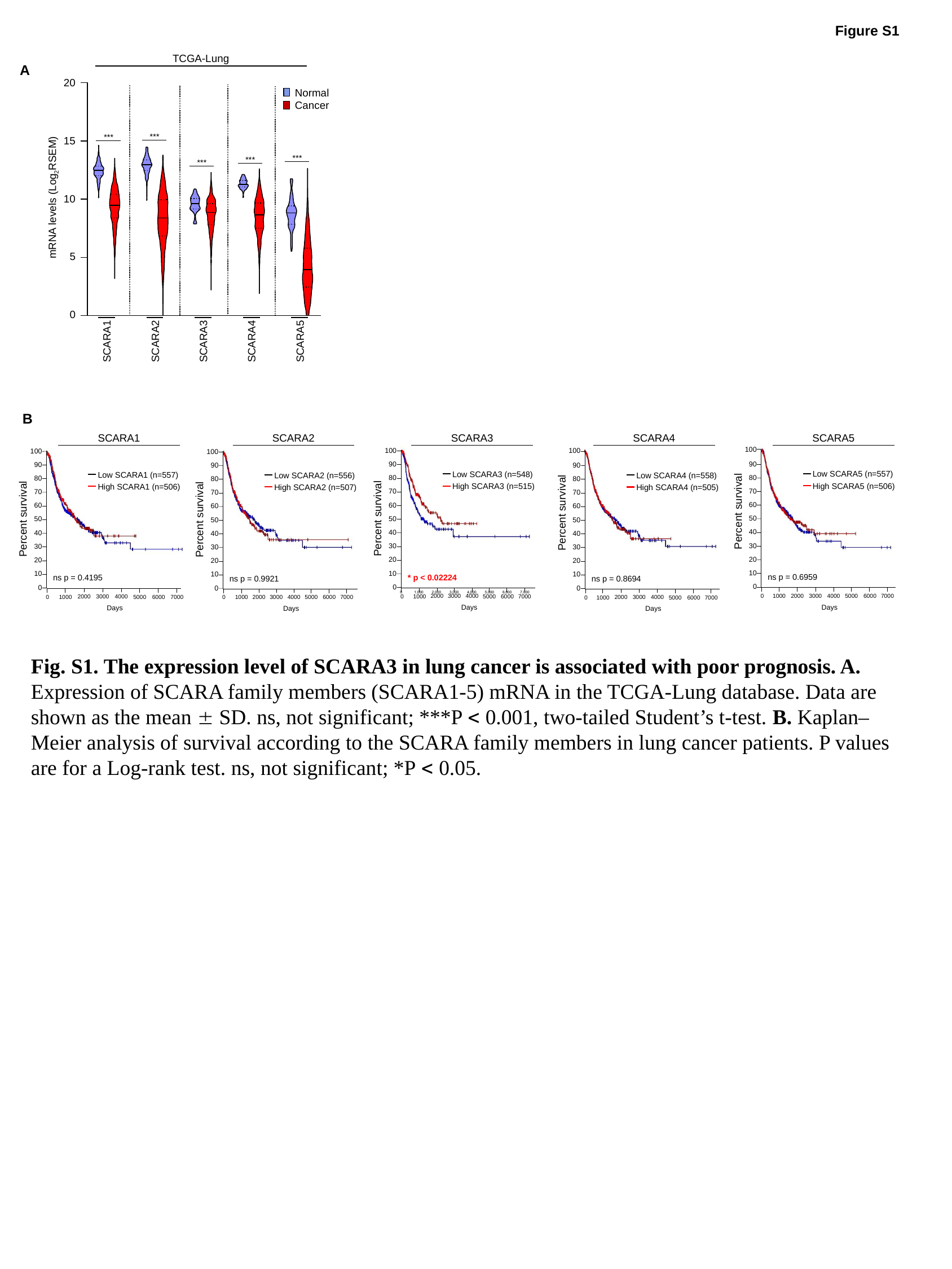

Figure S1
TCGA-Lung
A
20
Normal
Cancer
15
***
***
***
***
***
mRNA levels (Log2RSEM)
10
5
0
SCARA1
SCARA2
SCARA3
SCARA4
SCARA5
B
SCARA1
SCARA2
SCARA3
SCARA4
SCARA5
100
100
100
100
100
90
90
90
90
90
Low SCARA5 (n=557)
Low SCARA3 (n=548)
Low SCARA1 (n=557)
Low SCARA2 (n=556)
Low SCARA4 (n=558)
80
80
80
80
80
High SCARA5 (n=506)
High SCARA3 (n=515)
High SCARA1 (n=506)
High SCARA2 (n=507)
High SCARA4 (n=505)
70
70
70
70
70
60
60
60
60
60
Percent survival
Percent survival
Percent survival
Percent survival
Percent survival
50
50
50
50
50
40
40
40
40
40
30
30
30
30
30
20
20
20
20
20
10
10
10
10
10
ns p = 0.6959
* p < 0.02224
ns p = 0.4195
ns p = 0.9921
ns p = 0.8694
0
0
0
0
0
3000
4000
2000
0
1000
5000
6000
7000
3000
4000
2000
0
1000
5000
6000
7000
3000
4000
2000
0
1000
5000
6000
7000
3000
4000
3000
4000
2000
2000
0
1000
5000
6000
7000
0
1000
5000
6000
7000
Days
Days
Days
Days
Days
Fig. S1. The expression level of SCARA3 in lung cancer is associated with poor prognosis. A. Expression of SCARA family members (SCARA1-5) mRNA in the TCGA-Lung database. Data are shown as the mean ± SD. ns, not significant; ***P  0.001, two-tailed Student’s t-test. B. Kaplan–Meier analysis of survival according to the SCARA family members in lung cancer patients. P values are for a Log-rank test. ns, not significant; *P  0.05.

## Slide 2
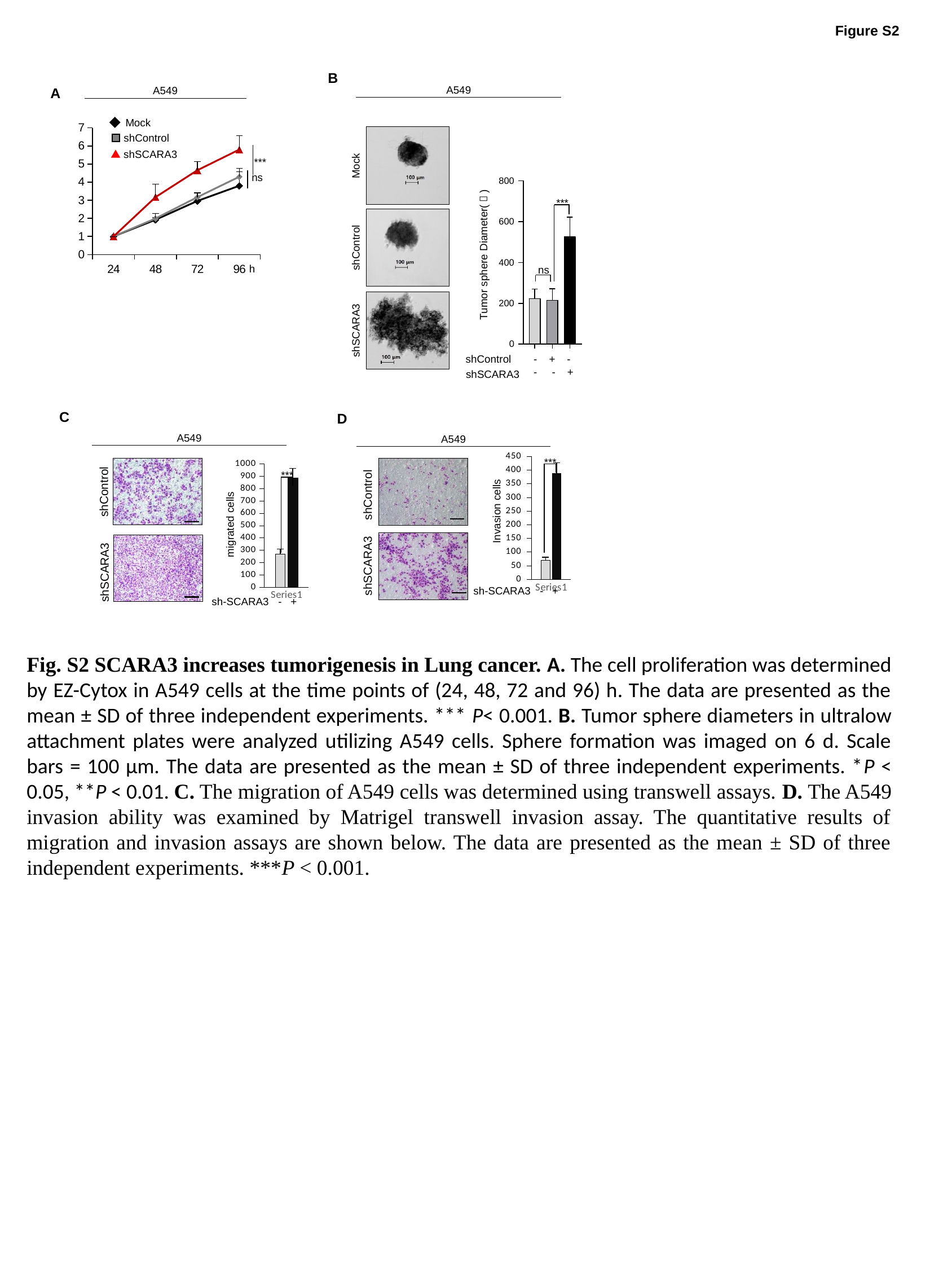

Figure S2
B
A
A549
A549
### Chart
| Category | A549-mock | A549-shControl | A549-shSCARA3 |
|---|---|---|---|
| 24 | 1.0 | 1.0 | 1.0 |
| 48 | 1.92 | 1.99 | 3.17 |
| 72 | 2.96 | 3.17 | 4.66 |
| 96 | 3.8 | 4.3 | 5.8 |Mock
shControl
shSCARA3
***
Mock
ns
***
shControl
Tumor sphere Diameter(㎛)
h
ns
shSCARA3
shControl
- + -
- - +
shSCARA3
C
D
A549
A549
***
### Chart
| Category | shControl | shSCARA3 |
|---|---|---|
| | 70.66666666666667 | 387.6666666666667 |
### Chart
| Category | shControl | shSCARA3 |
|---|---|---|
| | 267.1666666666667 | 885.3333333333334 |
***
shControl
shControl
Invasion cells
migrated cells
shSCARA3
shSCARA3
sh-SCARA3
- +
sh-SCARA3
- +
Fig. S2 SCARA3 increases tumorigenesis in Lung cancer. A. The cell proliferation was determined by EZ-Cytox in A549 cells at the time points of (24, 48, 72 and 96) h. The data are presented as the mean ± SD of three independent experiments. *** P< 0.001. B. Tumor sphere diameters in ultralow attachment plates were analyzed utilizing A549 cells. Sphere formation was imaged on 6 d. Scale bars = 100 µm. The data are presented as the mean ± SD of three independent experiments. *P < 0.05, **P < 0.01. C. The migration of A549 cells was determined using transwell assays. D. The A549 invasion ability was examined by Matrigel transwell invasion assay. The quantitative results of migration and invasion assays are shown below. The data are presented as the mean ± SD of three independent experiments. ***P < 0.001.

## Slide 3
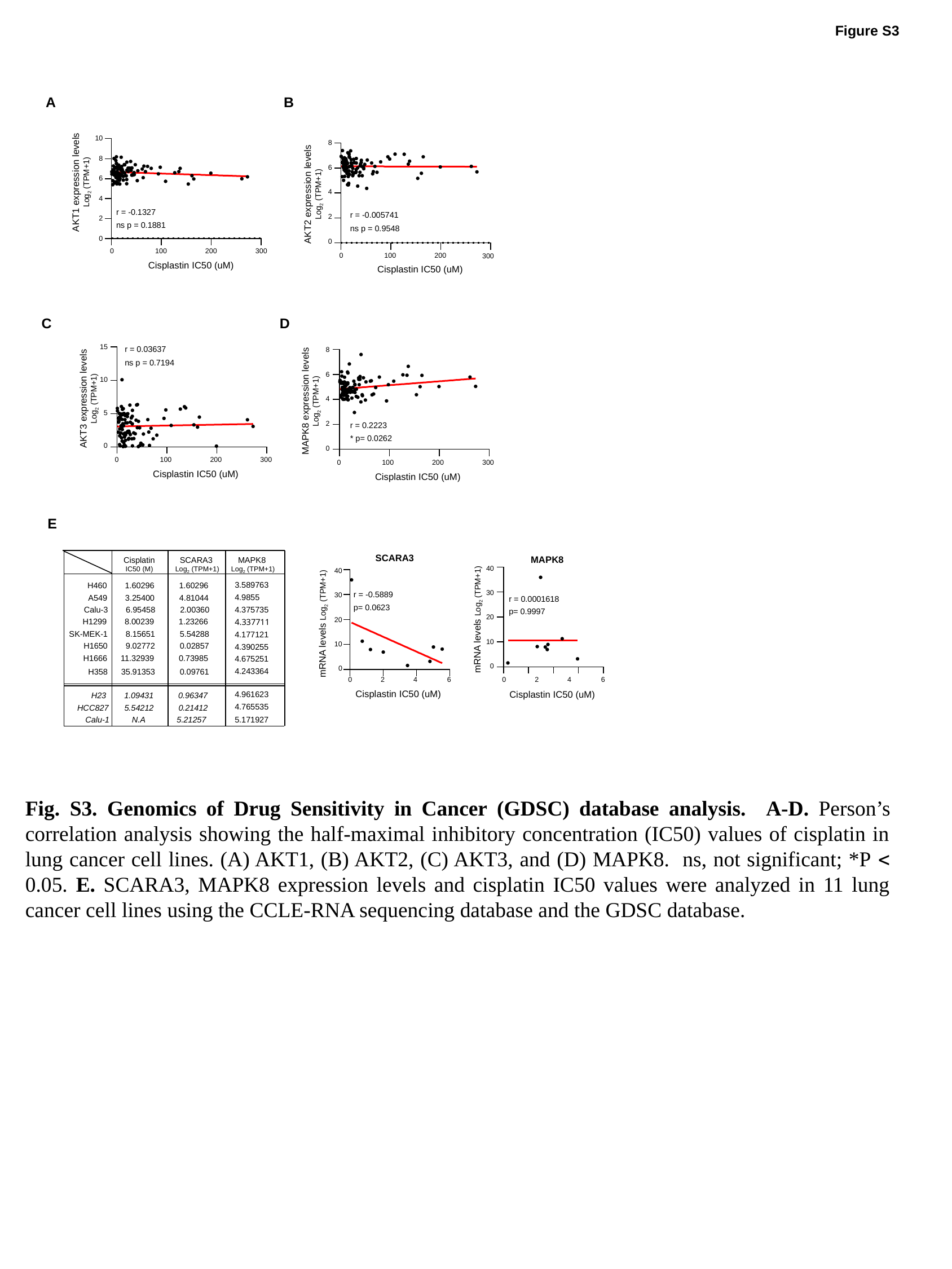

Figure S3
A
B
10
8
8
6
AKT1 expression levels
Log2 (TPM+1)
6
AKT2 expression levels
Log2 (TPM+1)
4
4
r = -0.1327
r = -0.005741
2
2
ns p = 0.1881
ns p = 0.9548
0
0
0
100
200
300
0
100
200
300
Cisplastin IC50 (uM)
Cisplastin IC50 (uM)
C
D
15
r = 0.03637
8
ns p = 0.7194
6
10
AKT3 expression levels
Log2 (TPM+1)
MAPK8 expression levels
Log2 (TPM+1)
4
5
2
r = 0.2223
* p= 0.0262
0
0
0
100
200
300
0
100
200
300
Cisplastin IC50 (uM)
Cisplastin IC50 (uM)
E
SCARA3
MAPK8
SCARA3
Log2 (TPM+1)
MAPK8
Log2 (TPM+1)
40
40
3.589763
H460 1.60296 1.60296
30
r = -0.5889
4.9855
30
A549 3.25400 4.81044
r = 0.0001618
4.375735
p= 0.0623
Calu-3 6.95458 2.00360
p= 0.9997
20
mRNA levels Log2 (TPM+1)
4.337711
20
H1299 8.00239 1.23266
mRNA levels Log2 (TPM+1)
4.177121
SK-MEK-1 8.15651 5.54288
10
4.390255
10
H1650 9.02772 0.02857
4.675251
H1666 11.32939 0.73985
0
4.243364
0
H358 35.91353 0.09761
0
2
4
6
0
2
4
6
4.961623
Cisplastin IC50 (uM)
Cisplastin IC50 (uM)
H23 1.09431 0.96347
4.765535
HCC827 5.54212 0.21412
5.171927
Calu-1 N.A 5.21257
Fig. S3. Genomics of Drug Sensitivity in Cancer (GDSC) database analysis. A-D. Person’s correlation analysis showing the half-maximal inhibitory concentration (IC50) values of cisplatin in lung cancer cell lines. (A) AKT1, (B) AKT2, (C) AKT3, and (D) MAPK8. ns, not significant; *P  0.05. E. SCARA3, MAPK8 expression levels and cisplatin IC50 values were analyzed in 11 lung cancer cell lines using the CCLE-RNA sequencing database and the GDSC database.
