# Supplementary figures and images for: SCARA3 inhibits cell proliferation and EMT through AKT signaling pathway in lung cancer
Source: BMC Cancer. 2022 May 16;22:552. doi: 10.1186/s12885-022-09631-z (PMC9112459; doi:10.1186/s12885-022-09631-z)

**Figure 1G**

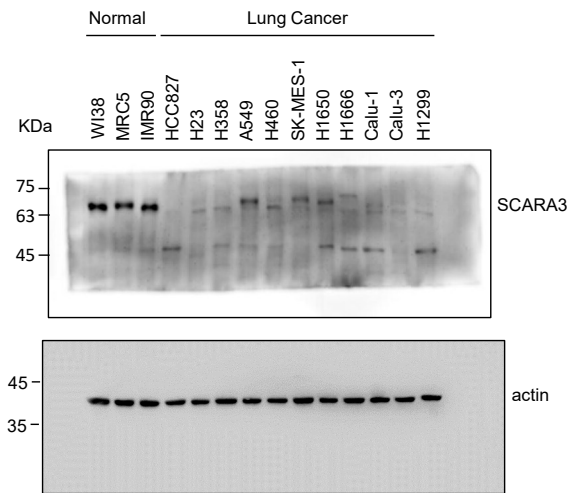

**Figure 3A**

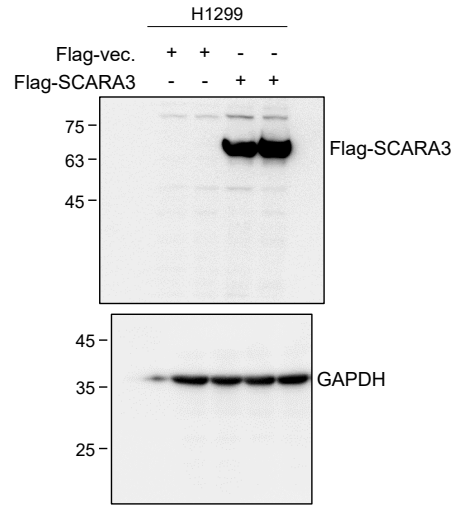

**Figure 3E**

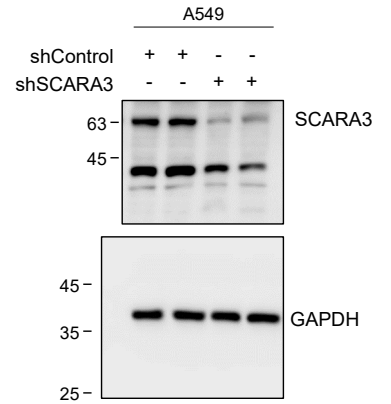

**Figure 4C**

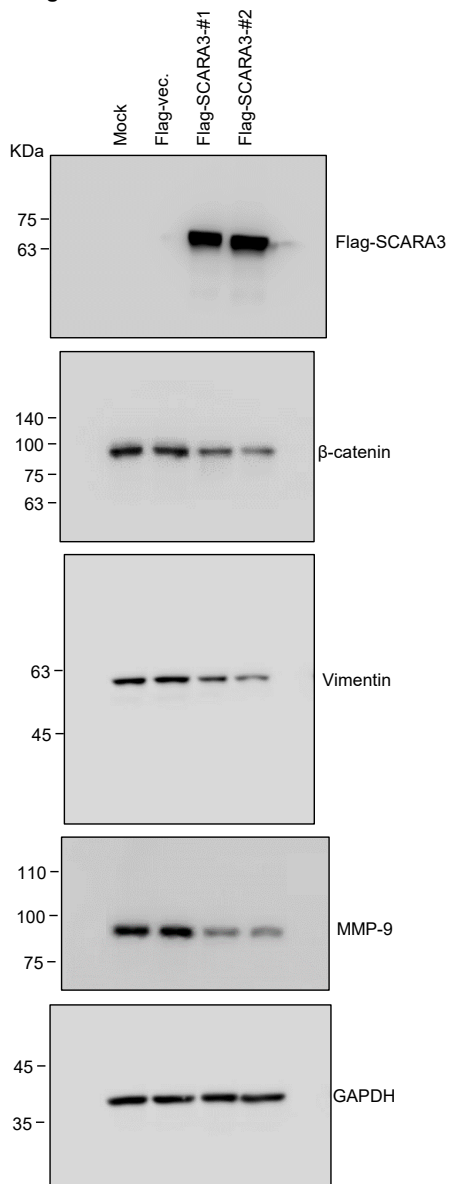

**Figure 4E**

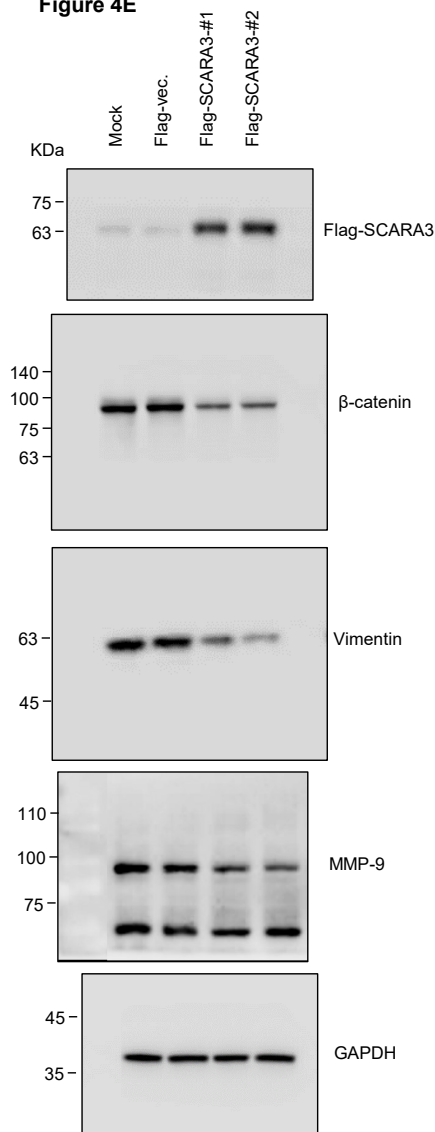

Figure 5C

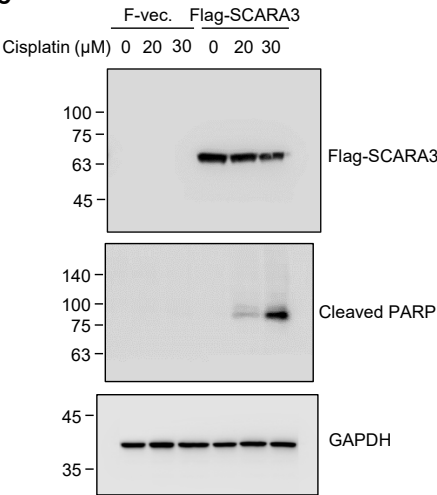

Figure 5F

|             |   |   |   |   |   |   |   |
|-------------|---|---|---|---|---|---|---|
| Flag-SCARA3 | - | - | + | + | + | + | + |
| SC79        | - | - | - | - | + | + | - |
| SP600125    | - | - | - | - | + | - | + |
| Cisplatin   | - | + | - | + | + | + | + |

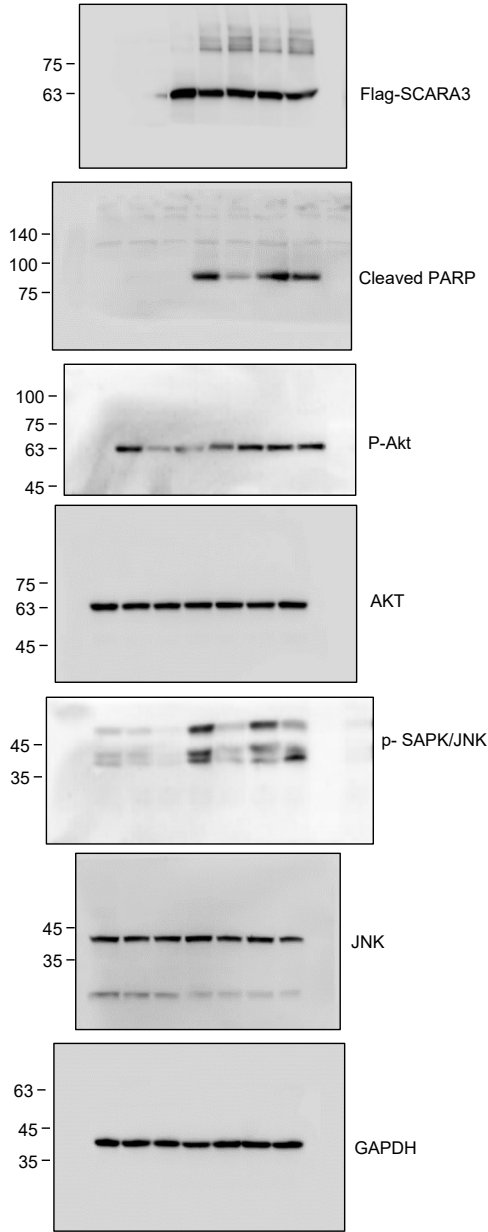

Figure 5D

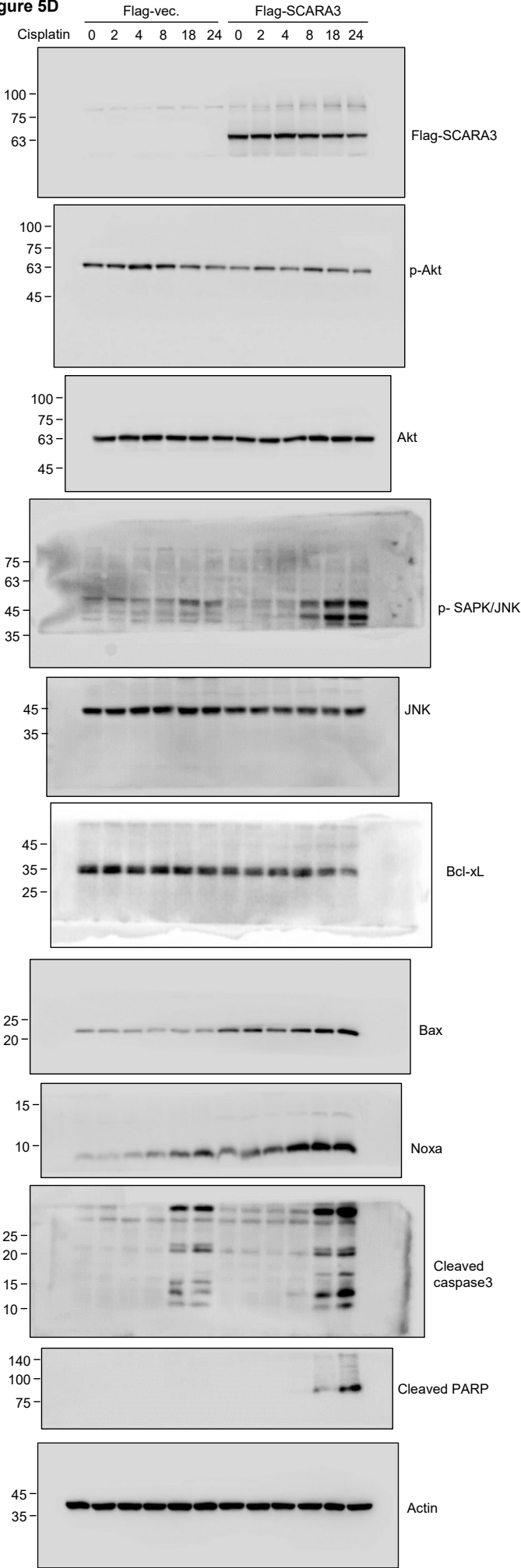

Supplement: Supplementary file 2 — Additional file 2. [file 12885_2022_9631_MOESM2_ESM.pdf]
